# P209: Governmental surveillance systems for healthcare-associated infection in the south and Southeast region of Brazil

**DOI:** 10.1186/2047-2994-2-S1-P209

**Published:** 2013-06-20

**Authors:** C Nogueira-Junior, MC Padoveze, RA Lacerda

**Affiliations:** 1 Nursing Department, Dr. Mario Gatti Hospital, Campinas, Brazil; 2 Public Health Department, Nursing School of University of São Paulo, São Paulo, Brazil; 3 Medical-surgical department, Nursing School of University of São Paulo, São Paulo, Brazil

## Introduction

A good surveillance is essential to gather information for measures to prevent and control of healthcare-associated infections(HAI). The study is aiming at characterize the HAI surveillance systems(HAI-SS) in 7 States in the South and Southeast region, of Brazil.

## Methods

Cross sectional and descriptive study carried out in two steps: characterization of healthcare system structure by means of consulting of the National Data Base of Healthcare Facilities; interview with person in charge of HAI program. HAI-SS were classified in chain, circle or wheel.

## Results

A large variation of healthcare facilities were identified. São Paulo is the State with highest number of healthcare facilities but the State of Santa Catarina has the highest ratio of healthcare facilities by 1.000.000 habitants(1,99). Human resources for HAI-SS varied, and, in some, they were not exclusive for data management but accumulated other functions. Hospital participation in the HAI-SS was mandatory by law in 3 States. HAI-SS were classified as chain in two, circle in four, and wheel in one State. HAI-SS were mainly driven toward acute care facilities; ventilator associated pneumonia, blood stream, urinary tract, and surgical site infections were included. Participation in the National HAI-SS occurred by sending data regarding blood stream infections. Routine feedback of surveillance data was not adopted in two States, one State have been using data gathered from HAI-SS to develop governmental plans for HAI rates reduction.

## Conclusions

It was identified inequalities in the healthcare services, potentially inducing to over crowding and posing to risk of low quality in some States. Human resources are insufficient in some States to carry out an adequate governmental plan for reduction of HAI rates. The operational dissimilarities among States may need to be overcome in order to build a good National HAI-SS.

## Disclosure of interest

None declared

